# Usefulness Of Ivabradine To Treat "unexpected" Heart Failure Caused By "acute" Right Ventricular Pacing

**Published:** 2011-10-02

**Authors:** Ermenegildo de Ruvo, Francesco Sebastiani, Luigi Sciarra, Alessandro Fagagnini, Leonardo Calo

**Affiliations:** UOC Cardiologia, Policlinico Casilino, Roma, ASL ROMA/B Italy

**Keywords:** Heart Failure, Ivabradine, sinus tachycardia, AICD, Atrioventricular Block

## Abstract

We present the case of a patient with a heart failure episode induced by acute right ventricular pacing. After reversal of beta-blockers because of chronic obstructive pulmonary disease (COPD) exacerbation, the following sinus tachycardia caused a 2:1 atrioventricular block and consequent continuous right ventricular pacing. He was treated with the selective I_f_ inhibitor ivabradine, that reduced both ventricular pacing percentage and heart rate without affecting atrioventricular conduction. Ivabradine may be a valuable option in treatment of patients with atrioventricular conduction disturbances.

## Introduction

Congestive heart failure is a common health problem. The burden of this condition is likely to increase as a result of an aging population and improved survival rates after acute myocardial infarction. In patients with symptomatic chronic heart failure, beta-blockers reduce mortality and hospital admission for worsening heat failure (Class I, level of evidence A) [1]. However, many heart failure patients still do not receive a beta-blocker or receive a suboptimal dose, because side effects may limit their use. Both COPD and atrio-ventricular conduction disturbances may forbid dosage up-titration.

## Case Report

In January 2010, a 79-year-old man with a ten year history of post-ischaemic dilated cardiomyopathy was admitted with dyspnoea at rest, i.e. NYHA functional class IV, after he reduced bisoprolol dose because of COPD exacerbation. He had received a dual chamber ICD (Epic DR V- 239, St. Jude Medical Inc, USA) in July 2009 because of an episode of sustained ventricular tachycardia. Coronary artery disease progression had been ruled out by angiography six months before. Apart from beta-blocker, he received a standard heart failure medication (including ACE inhibitor, digitoxin, furosemide and spironolactone). On physical examination, a grade 2 holosystolic murmur at the left axillary's border at pulmonary auscultation basal rales were detected, blood pressure was 120/80 mmHg, and pulse was regular (105 bpm). Physical examination was otherwise unremarkable. The ECG showed sinus rhythm, with right ventricular pacing and a heart rate at 105 bpm (Figure 1A).

**Figure 2:**
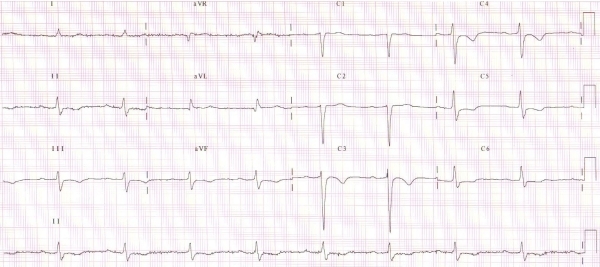
ECG at discharge: sinus bradycardia without right ventricular pacing

Chest X-ray demonstrated cardiomegaly with signs of pulmonary congestion. Laboratory test results revealed elevated but stable values of BUN (96 mg/dl) and creatinine (2.01 mg/dl). NT-pro-BNP was 970.9 pg/ml. All other laboratory parameters including thyroid function were normal. Echocardiography demonstrated left ventricular (LV) dilation with an ejection fraction of 39 % calculated by the Teichholz' method. Color Doppler imaging demonstrated mitral regurgitation grade 3. At ICD interrogation 2:1 AV block with a narrow QRS was recognized (Figure 1B). We hypothesized a frequency dependent AV block. Thus, to slow heart rate the patient started the selective If channel inhibitor ivabradine at the dose of 5 mg bid, bisoprolol was stopped. The patient reported a prompt and marked symptomatic improvement. ECG showed sinus rhythm, with 1st degree AV block and left anterior hemiblock, without RVP (Figure 2). At noninvasive pacing stimulation (NIPS) a 2:1 AV conduction block was recorded with a drive of 600 ms. Three days after admission the patient was asymptomatic at rest and was discharged with a resting heart rate of 51 bpm. Echocardiography showed a slight increase of the left ventricular ejection fraction to 45%, and a reduction of mitral regurgitation to grade 2. Therefore, due to unexpected functional improvement, adequate heart rate control and reduction of right ventricular pacing he was discharged. He is now in stable NYHA class II. No adverse effects to ivabradine treatment have been reported by the patient.

## Conclusion

Faster resting heart rates represent per se a risk factor for cardiovascular mortality and HF hospitalization, despite optimal HF therapy. Heart rate may condition left ventricular filling, myocardial oxygen demand and coronary perfusion time. [2,3] Beta-blockers remain the therapy of choice in all patients with systolic heart failure, but they may worsen AV conduction and increase RV pacing percentage. Nevertheless, reduction of cumulative RV pacing as far as possible should be achieved in ICD patients. Several studies have demonstrated detrimental effects of right ventricular (RV) apical pacing on cardiac function [4,5]. The direct electric stimulation of the RV apex causes an abnormal activation sequence and mechanical dyssynchrony. In patients with LV dysfunction, these effects are more pronounced and permanent RV apical pacing may cause a higher risk of morbidity and mortality at long-term follow-up.

Sharma et al. showed in a post hoc analysis of the DAVID trial that DDDR RV pacing >40% was a univariate predictor of death and hospitalization for heart failure [4]. Smit et al. studied the effect of RV pacing on heart failure events in asymptomatic (NYHA class I) patients. Cumulative RV pacing >50% was associated with an increase in heart failure events and was an independent predictor of ICD shocks [5].

Ivabradine is a specific inhibitor of the If current of the sinus node, that induces a selective and dose dependent HR reduction pure HR lowering agent without effects on atrioventricular conduction or contractility. The SHIFT study has recently assessed that heart rate reduction by direct sinus node inhibition can reduce cardiovascular mortality or HF hospitalization by 18% in patients with chronic heart failure and left-ventricular systolic dysfunction. In particular, this beneficial effect was mainly driven by a favourable effect on HF death/hospital admission (RRR 26%) [6] This case demonstrates that RV pacing may be reduced by controlling heart rate at rest by using ivabradine. Pure heart rate reduction without effect on AV conduction obtained by ivabradine may be a valuable option in order to avoid increase in RV pacing percentage in dual chamber ICD and pacemaker recipients when sophisticated algorhithms are not available or drug induced atrio-ventricular block may be detrimental. Further studies are needed to evaluate the use of Ivabradine to control HR in dual chamber pacemaker and ICD recipients with moderate to severe left ventricular dysfunction and atrio-ventricular conduction disturbances in order to obtain HR control without promoting right ventricular pacing.

## Figures and Tables

**Figure 1A:**
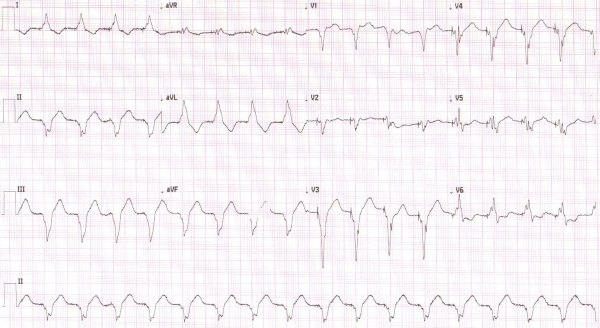
ECG at admission: sinus tachycardia with right ventricular pacing.

**Figure 1B:**
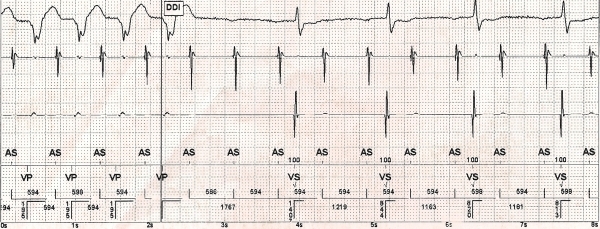
ICD interrogation at admission reveals a 2:1 atrio-ventricular block.
